# Effect of a Single Intrauterine Dose of Human Recombinant Galectin-1 Buffered on Pregnancy Rate in Inseminated Cows

**DOI:** 10.3390/biom12030419

**Published:** 2022-03-09

**Authors:** Erika da Silva Carvalho Morani, Helen Alves Penha, Fernando Sebastián Baldi Rey, Marcelo Roncoletta

**Affiliations:** 1 Yoni Group, Inprenha Biotecnologia, Jaboticabal 14870-970, São Paulo, Brazil; emorani@inprenha.com.br (E.d.S.C.M.); hpenha@inprenha.com.br (H.A.P.); 2 Departamento de Zootecnia, Faculdade de Ciências Agrarias e Veterinárias (FCAV), Universidade Estadual Paulista (UNESP), Jaboticabal 14884-900, São Paulo, Brazil; fernandobaldiuy@gmail.com

**Keywords:** Tolerana^®^, fetal-maternal recognition, mammals reproduction, efficacy test

## Abstract

The objective was to evaluate the efficacy of a single dose of exogenous galectin-1 in improving the pregnancy rate in inseminated cows, comparing the pregnancy rate of the two groups (treatment and control Groups) into 107 contemporary groups (YG) established. An ultrasound exam determined the pregnancy rate performed 25 to 35 days after the fixed-time artificial insemination (FTAI) of breeding beef cows (n = 3469). The pregnancy rate of cows that received a single dose of eGAL-1 (200 ± 10 µg), with an intrauterine administration (n = 1901), was compared with the pregnancy rate of cows inseminated using a conventional AI protocol (n = 1568), both comparing into the same YG. YGs were created considering the grouping of cows belonging to the same farm, with the same nutritional score and management, inseminated by the same inseminator and semen batch, and using the same estrus synchronization protocol). The statistical method used calculated the probability of obtaining pregnancy within each group. The administration of a single dose of eGAL-1 can increase the probability of obtaining pregnancy in beef cows by up to 8.68% (*p* < 0.0001), suggesting that a single dose of eGAL-1 during the FTAI procedure was reasonable in the beef cattle AI routine and can improve the pregnancy rate considerably.

## 1. Introduction

Galectin-1 (GAL-1) is implicated in maternal-fetal tolerance, associated with regulating and modulating the immunological responses, embryo elongation events, and endometrium adherence. It contributes to placentation, development, migration, and trophoblastic invasion, essential in early gestational development. GAL-1 has been cited as a mediator in preventing early embryonic death in mammals. Because of that, since 2008, the authors have worked on the hypothesis to use an effective dose of human recombinant galectin-1 as a tool to improve the efficiency of reproductive biotechnologies procedures, such as artificial insemination (AI, FTAI) or embryo transfer (ET) in different species (bovine, goats, sheep, and equine). Thinking of the future possibility of using eGAL-1 administration in human reproduction procedures, we are spending efforts to comprehend its pharmacology, and this article is part of them. The objective of this study was to determine the impact of using a single intrauterine dose administration of eGAL-1 on the pregnancy rate. An efficacy test using bovine females (beef cows) as a model can also help demonstrate the security to use this interspecies protein and confirm this as an exciting tool to improve reproduction performance.

Why was GAL-1 a choice? Several causes can explain the reduction in the pregnancy rate, including early embryonic mortality [1], diseases [2], uterine asynchrony inducing failure of maternal recognition of pregnancy [3,4], nutrition and milk production [5], placental homeostasis, and uterine environment [4,6], embryo-lethal genetic mutations [4], and unbalanced immunological factors at the maternal-fetal interface [7]. Complex immunoregulatory mechanisms at the maternal-fetal interface must be balanced to activate maternal tolerance against fetal alloantigens and protection against infections and inflammation [8]. Many mediators are involved in these mechanisms, and galectins, including GAL-1, play a crucial role, generating significant interest in reproductive medicine, due to their unique ability to modulate various processes of gestational development and their potential use biomarkers for gestational disorders [9].

The way to practice the hypothesis was to produce a recombinant human GAL-1 to be tested on different mammals’ species (that explains the name “exogenous GAL-1” created- eGAL-1). It was based on the high degree of structural conservation, dimerization, and binding properties with carbohydrates and integrins, suggesting that these properties are conserved among vertebrates and maintain a gene expression pattern among the different types of the placenta (deciduous or not) [10].

The bovine species was the first choice to measure the efficacy of a single intrauterine dose of eGAL-1 because of the possibility of using many inseminated females and the significant facility of administration via (intrauterine administration). As complementation of literature, this article remarkably elucidates the effect of eGAL-1 on reproductive physiology. It positively impacts the development and maintenance of pregnancy in cows (efficacy) and exemplifies good pharmacological safety, allowing for clinical tests in humans.

## 2. Material and Methods

### 2.1. Study Design

This study analyzed the increase in the pregnancy rate by administering a dose of exogenous GAL-1 (eGAL-1), combined with the technique of fixed-time artificial insemination (FTAI) in beef cattle. An effective dose of eGAL-1 means 1 (one) dose of Tolerana^®^ (Inprenha Biotecnologia, Jaboticabal, Brazil) whose administration is “extra,” but similar to the application of a dose of semen, during the FTAI procedure. One effective dose of eGAL-1 contains 200 ± 10 µg of human recombinant protein (rHGAL-1), diluted in 200 µL of sterile PBS 1X pH 7.0 buffer solution (phosphate-buffered saline with kanamycin sulfate) present in a 0.25 mL French-style straw. The definition of what represents an effective dose of eGAL-1 was established in previous experiments (unpublished data), where five different doses were tested, always considering use in the same presentation and administration model cited in the present experiment.

The effective dose was established by analyzing the relationship between “the lowest protein dose” versus “desired effect on increased pregnancy rate” in beef cows. Doses of 200 µL containing 0, 50, 100, 150, 200, and 300 µg of eGAL-1 were tested in a unique lot (Nellore cows, in one farm, inseminated by one technician, using one semen batch). Those amounts of protein were tested, thinking in the area (cm^2^) of the cow’s lumen uterus. A group of 300 cows, subdivided into 50 cows for each dose group, were tested. Groups of animals that received doses of 50 and 100 µg did not show a significant increase in pregnancy rate compared with the control group results. The group that received 150 µg had an increase in pregnancy rate compared with the control group. However, the increase was more evident in the groups that received doses bigger than 200 µg (13 percentage points compared to the control group). It was decided to follow the efficacy tests with a dose of 200 µg per AI procedure in cows, considering cost-effectiveness.

rHGAL-1 was obtained by constructing a heterologous expression vector containing the gene (pET-29a(+)+lgals-1 gene) and purification to obtain active, sterile protein in its alkylated form and free of endotoxins.

The efficacy of eGAL-1 was verified by comparing the pregnancy rate in bovine females combining the administration of semen and eGAL-1 (treatment group—TG) versus the administration of only one dose of semen (control group) in the procedure of TFIA. Thus, in TG, the eGAL-1 dose is deposited in the uterus lumen after the semen dose deposition. Therefore, there are two procedures for passing the applicators through the cervix. Pregnancy rates for each group (TG and CG) were determined by ultrasound diagnosis (between 28 and 35 days after the FTAI procedure) and submitted to statistical analysis (described on Section 2.5).

### 2.2. Galectin-1 (GAL-1) Production and Purification

GAL-1 can be obtained from mammalian genomes (from species such as human, bovine, ovine, caprine, equine, and porcine) through heterologous expression systems. It was performed by preparing GAL-1 solutions as an active protein sterile, alkylated, and free of endotoxins. The method for obtaining human recombinant Galectin-1 is determined Yoni Group/Inprenha Biotecnologia^®^ and involves the following steps: (i) obtaining a crude extract of bacteria cultivated to express GAL-1; (ii) purification of Galectin-1; (iii) preservation of the lectin activity of Galectin-1 by alkylation; (iv) removal of bacterial endotoxin (LPS) from the alkylated Galectin-1 solutions; (v) adjustment of protein concentration; (vi) filling and (vii) quality control. Among the possibilities disclosed in the literature for the upstream and downstream steps, Galectin-1 was produced based on the following procedures, including particularities of the manufacturer’s process.

#### Subcloning of Gal-1 into pET-29a(+) Expression Vector

Gal-1 Consensus Coding Sequence (CCDS) CCDS13954.1 (length 408 nt) was synthesized and subcloned, with juxtaposed insertion of the desired sequence, immediately after the RBS Ribosome binding site sequence of a pET-29a(+) expression vector cut in NdeI/HindIII (GenScript^®^, Piscataway, NJ, USA). This construct was then used for competent transformation of the Rosetta strain of Escherichia coli, maintained in a cell bank.

### 2.3. Bacterial Culture, Expression, and Lysis

Aliquots of *E. coli* strains transformed with the vector insertion containing the GAL-1 gene (pET-29a(+)+lgals-1 gene) were grown in systems with LB Broth Base medium containing kanamycin sulfate until obtaining optimal bacterial growth rate, demonstrated by optical density. Induction of expression is performed by adding 0.3 mM Isopropyl-d-Thiogalactopyranoside (Sigma-Aldrich, I6758, Burlington, MA, USA) to the culture. After the induced growth period (4.5 hs), the bacterial suspension is retained by microfiltration on a hollow fiber membrane (0.22 µm, Cytiva, CFP-2-E-9A, Marlborough, MA, USA) and centrifuged at 5000× *g* for 15–20 min at 4 °C, always with the supernatant being discarded and the “bacterial crude = pellet” which were then subjected to bacterial lysis.

For Bacterial lysis, the crude or bacterial pellet was resuspended in phosphate saline lysis buffer (1× PBS—136.8 mM NaCl, 2.7 mM KCl, 6.4 mM Na_2_HPO_4_, 0.9 mM KH_2_PO_4_, pH 7.4), containing 14 mM Mercaptoethanol, protease inhibitor EDTA-free, lysozyme-1, RNAse A-Type 3A, and DNAse I Type IV-10. All components are Sigma-Aldrich. The pellet diluted in lysis buffer (Chemical Lysis) was subjected to constant homogenization for 70 min and then sonicated for three cycles of 15 s each in a Vibra-Cells Sonicator, Sonics (Mechanical Lysis), with intervals of 20 s between each cycle. The bacterial lysate was then clarified by centrifugation at 7000× *g* for 20 min at 4 °C and filtered through a 1.0 µm filter (Whatman) with the aid of a peristaltic pump (maximum pressure of 4 BAR).

### 2.4. Purification Steps

After the chemical and mechanical lysis process, the lysate was submitted to 3 steps of purification by chromatography in an AKTA Protein Purification System (Cytiva) to obtain a buffered protein solution containing only Galectin-1.

The first step is based on affinity chromatography on agarose-lactose columns (Sigma-Aldrich), previously equilibrated with equilibration buffer (1× PBS, 14 mM 2-ME, pH 7.4). After injection of the protein solution, the affinity column “binders” were washed and eluted with elution buffer (1× PBS containing lactose and 2-ME pH 7.4). The protein peak was collected, and 20 μM of iodoacetamide (Sigma-Aldrich; I1149) was added to the solution, keeping it under incubation at 4 °C, protected from light, and overnight. After this incubation, the solution was subjected to “size exclusion” chromatography (Sephadex G-25, Cytiva) to remove the free salts of iodoacetamide and lactose. The last chromatographic step was the removal of bacterial endotoxins (LPS). To this end, the preparations were subjected to chromatography using LPS affinity resin (PIERCE High-Capacity Endotoxin Removal Resin column—Thermo Scientific, 88270, Waltham, MA, USA). After all the chromatographic steps, the protein concentration was determined by spectrometry (Abs 280 nm) and expressed in milligrams of protein per milliliter (mg/mL) and was submitted to sterilizing filtration (0.22 μm PES membrane).

Purified protein batches were submitted to quality control to check the following: determination of protein concentration; microbiological status; protein bioactivity (Hemagglutination test); molecular weight analysis by SDS-PAGE; and SEC (size exclusion chromatography; protein secondary structure analysis (Circular Dichroism Analysis); aggregate detection and molecular size by DLS (Dynamic Light Scattering) analysis and endotoxin quantification (LPS). Protein identity was confirmed by LCMS (liquid chromatography-mass spectrometry) and nucleotide sequence confirmation of human galectin-1 cDNA-galectin-1 [Homo sapiens] Consensus Coding Sequence (CCDS) CCDS13954.1 (https://www.ncbi.nlm.nih.gov/projects/CCDS/CcdsBrowse.cgi?REQUEST=ALLFIELDS&DATA=CCDS13954.1&ORGANISM=0&BUILDS=CURRENTBUILDS, with the last access at 18 August 2020).

### 2.5. Field Experiment

#### 2.5.1. Location

The experiments were conducted in 17 commercial beef cattle farms located in different Brazilian municipalities (Cuiabá/Mato Grosso; Campo Grande, Naviraí and Água Clara/Mato Grosso do Sul; Formoso do Araguaia and Gurupi/Tocantins; Paragominas/Para; Uberaba, São Gotardo and Prata/Minas Gerais; and Pedregulho/São Paulo). It should be noted that the farms selected to participate in this experiment were farms that have a history of working with FTAI procedures for at least two years.

#### 2.5.2. Animals

**Figure 1:**
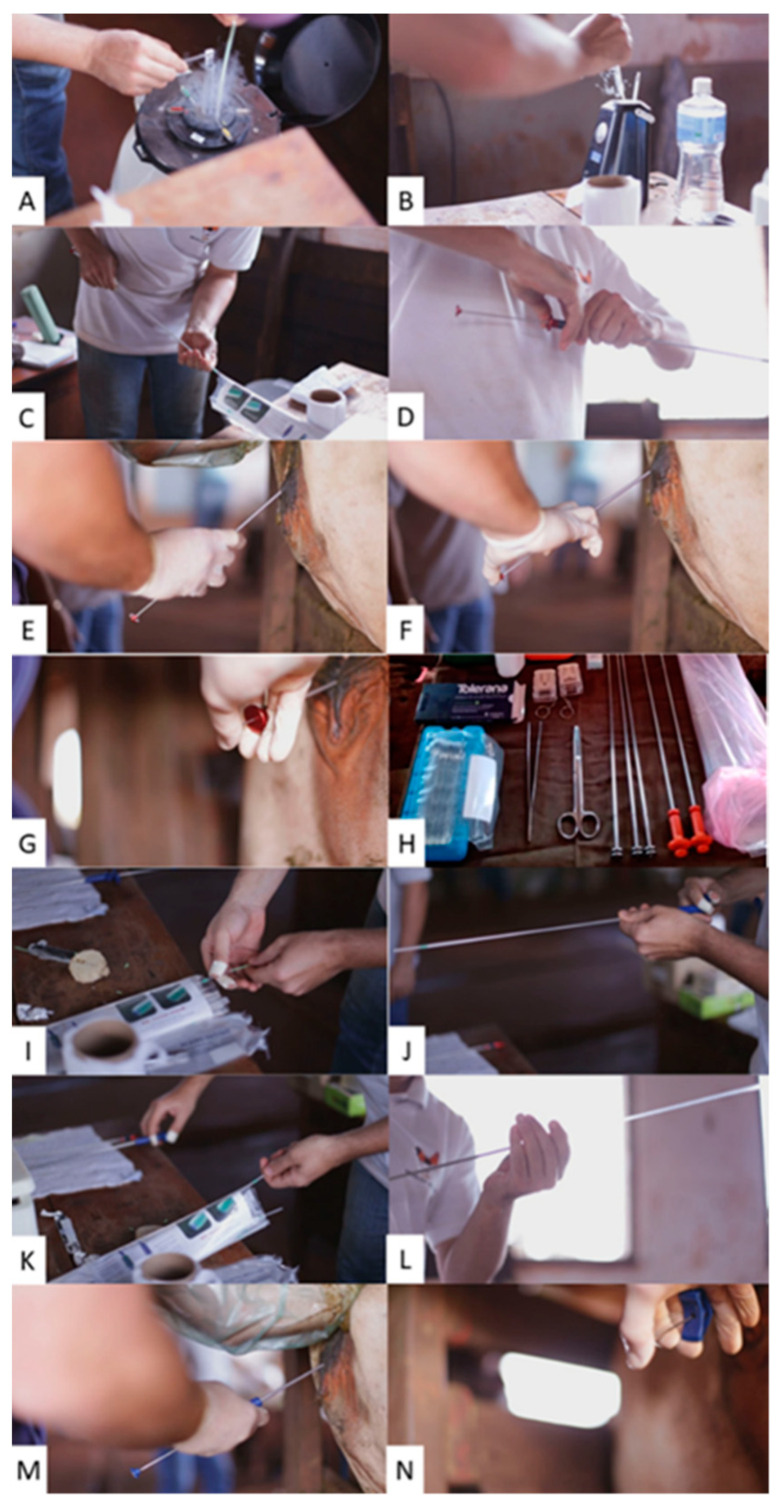
Procedure for the administration of Tolerana Bovines IA. in sequential acts, they follow illustrations of the following steps: (**A**) removal of the semen reed from the nitrogen canister; (**B**) semen thawing; (**C**,**D**) assembly of the applicator with a semen reed; (**E**) passage of the applicator through the cervix; (**F**,**G**) application of semen to the body of the uterus (**H**) cut the end of Tolerana’s reed; (**I**–**L**) assembly of the applicator with Tolerana IA reed; (**M**) passage of the applicator through the cervix; (**N**) application of Tolerana Bovines IA in the body of the uterus.

The experiments were carried out on female bovine animals (cows conventionally managed as dams and not intended for slaughter) managed in extensive beef cattle rearing systems. The dams were kept in an extensive rearing system, under native and/or cultivated pasture, with mineral supplementation. All cows underwent an FTAI procedure. It should be noted that only cows diagnosed as empty (by ultrasonography, 28 to 35 days after the first service—1st FTAI) were worked on in a second FTAI protocol (Figure 1). Fifteen days from the 2nd. FTAI, bulls were introduced for transfer with natural breeding. It is important to remember that the experiment and the statistical model considered only the “first service” results to ascertain the effectiveness of the dose of eGAL-1. Before the breeding season started, some farms implemented prophylactic management with annual vaccinations against BHV-1, BVDV, and BL (dose and booster).

In total, 3469 beef cows (Nellore and crossbred dams) were considered in the statistical model, which were divided into two treatment groups (TG and CG) and for statistical analysis divided into 107 contemporary groups (YG) as described below. The experiment was designed with 4730 dams at the time of insemination, distributed equally (same number of cows in each group n = 2365) and randomly (without the previous choice of the TG or CG group that would be part of). However, 1261 cows were excluded from the experiment for different reasons, including: (i) dams which did not maintain BSC between 3.5 and 2.5; the dams’ body condition score (BSC) was observed in two situations—at the time of the FTAI and the day before the pregnancy diagnosis; only dams that maintained a BSC between 3.5 and 2.5 in the two situations mentioned above were approved to participate in the statistical analysis; (ii) dams which died during this interval; (iii) dams which became ill during this interval (e.g., hoof, mastitis, diarrhea and pneumonia); (iv) dams which had problems with the synchronization protocol (e.g. loss of CIDR); (v) dams which changed management lot). For these reasons, it is noted that, in some farms, the number of dams mentioned in Table 3 differs between the TG and CG groups. The numerical decompensation between groups was corrected in the construction of contemporary groups (YG), as described in Section 2.5.5. below. An important detail is that the dams were submitted to BSC classification before the pregnancy diagnosis.

The criteria for defining the used BSC were based on the descriptions by Machado et al. (2008) [11], who empirically determined the separation of dams into 5 BS classifications: 1 (cachectic): complete visualization of the ribs, exposures of ileum bones and ischium, and pronounced muscle atrophy (skin and bones apparent); 2 (thin): very prominent bones with visible dorsal, iliac and ischial processes; 3 (great): light muscle coverage and no fat accumulation; 4 (fat): good muscle coverage and fat deposition at tail insertion; 5 (obese): all body angles covered, including protruding skeletal parts and overall animal appearance.

The experiment considered three different animal categories in the work lots—heifers, multiparous and primiparous. Multiparous and primiparous cows had calves on their feet at 60 to 100 days of lactation. These categories defined differences in the estrus synchronization protocols used for the categories.

#### 2.5.3. FTAI and eGAL-1 Administration

The breeding cows were kept in management batches on the farms. Each batch was submitted to FTAI after estrus synchronization protocols. These synchronization protocols were decided by each farm, as described in Table 1. No interference was imposed on farms in (i) the choice of estrus synchronization protocols, (ii) regarding the selection of the “bull” (semen doses), and (iii) the choice and training of the inseminator who would inseminate each batch of breeding cows. A total of 46 bulls were used, selected by the partner farm, and the semen doses of each bull were distributed in the treatment (TG) and control (CG) groups. In total, 23 inseminators participated in the experiment carried out on these 17 farms. There was no previous selection for the dam to receive the dose of eGAL-1 during the AI procedure.

The procedure in the treated group TG was to inseminate the breeding cows using a conventional semen applicator, followed by the administration of the eGAL-1 dose. For that, it used a second applicator (identical to the semen), which represents that breeding cows in the treated group were at a disadvantage compared to the CG, as they had two events of the transgression of the cervical rings. The deposition of the eGAL-1 dose in the uterine lumen was performed as the second insemination where, after removal of the semen applicator, a second applicator mounted with a straw containing the protein dose was re-introduced.

As a procedure in the CG, the females were inseminated according to the standard procedure, the same one recommended by [12] (Brazilian Association of Artificial Insemination, 2018), with a single dose of semen (which represents one applicator being passed through the cervical rings). The time spent for the insemination procedure in the females of the CG and TG was observed and noted. In this experimental model, we worked with the prerogative that dams belonging to the CG were at an advantage compared to the TG, as they received only “one-act” to transverse the cervical rings during the AI procedure in cows, and that advantage to the pregnancy rate to CG.

#### 2.5.4. Obtainment of the Pregnancy Rate in the Groups

The statistical methodology calculated the increase in the pregnancy rate obtained using the eGAL-1 considering dams, which maintained a BSC between 3.5 and 2.5, divided into two experimental groups (TG and CG) compared within the same contemporary groups formed, as described below. A technician performed the diagnosis with experience in ultrasonography and without knowledge of the division of dams into the TG and CG.

#### 2.5.5. Contemporary Groups and Statistical Analysis

To define and compare the pregnancy rates between breeding cows inseminated with and without a single dose of eGAL-1, they were grouped into contemporary groups (YG). Each contemporary YG 3 was composed of dams inseminated by: the same inseminator (identified by a letter code); belonging to the same farm (identified by a letter code); belonging to the same animal category (M = multiparous, P = primiparous or H = heifers); belonging to the same management group (identified by the FTAI date + Farm code + management lot code); inseminated with the same semen batch (identified by the name of the bull) and being of the same breed (N = Nellore and CB = crossbreed). A minimum number of at least five dams was considered to form a YG, or those groups that did not show variation in the pregnancy rate (100 or 0%) were also discarded. The generalized linear model (GLM) was applied to perform the analysis, with the GENMOD procedure of the SAS Institute (2009)—SAS Proprietary Software Version 9.3, SAS Institute, Cary, NC, assuming a binomial distribution (pregnant or not pregnant) with residual effect and a logarithmic function (PROBIT). The model included the fixed effect of YG and treatment (dose = 0 of eGAL-1 in the CG and dose = 200, which means 200 ± 10 µg of eGAL-1 in the TG).

Altogether, 107 YGs were formed, considering these 3469 breeding cows, distributed among the three animal categories (heifer, primiparous, and multiparous), considering 46 different bulls’ semen, and 23 different inseminators and, many management batches. Remember that 1261 dams were excluded from the statistical analyses, as mentioned previously.

The PROC GENMOD is modeling the probability of the pregnant rate, using effect parameters (dose 0, dose 200, YG, and interception). Dose 200 means administration of eGAL-1 in TG dams. The pregnancy rate obtained in these 107 YG was determined considering pregnancy diagnosis by ultrasound between 28 and 35 days after the FTAI procedure.

## 3. Results

**Figure 2:**
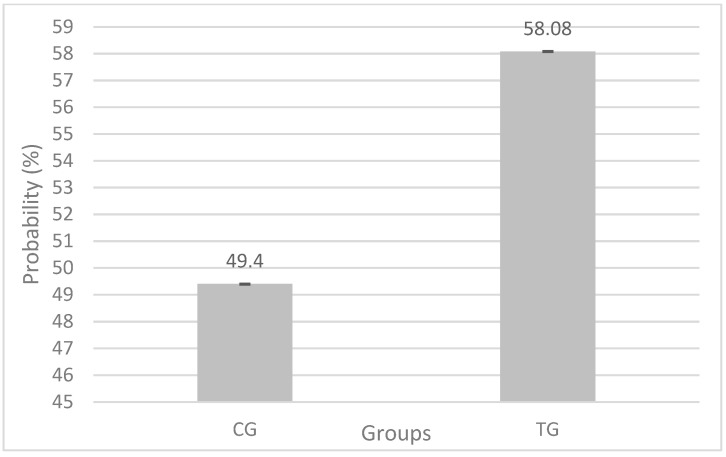
Probability of obtaining a positive pregnancy in the control (CG) and treatment (TG) groups when compared within the YG, according to the statistical model used.

In total, 3416 cows distributed in 107 YG were considered on statistical analysis, 1515 in the CG and 1901 in TG. The administration of a single eGAL-1 dose during the FTAI procedure took about 10 ± 5 s longer than a conventional procedure. The probability of positive pregnancy in the CG group was 49.4%, while in the TG group (n = 1919) it was 58.08% (*p* < 0.0001), as detailed in Table 2. The mean obtained with dose 0 = 0.491, equivalent to 49.41% of probability of obtaining a positive pregnancy in the CG, while the average obtained with dose 200 = 0.5808, equivalent to 58.08% probability of obtaining a positive pregnancy in the TG (Figure 2), which represents 8.68% difference between the treatment groups when compared within of each YG. The “YGs effects,” under binomial distribution (“pregnant” and “not pregnant”), did not present statistical significance (*p* = 0.1787), perhaps because the variables that made up the construction of the YG significantly interfere in the pregnancy rate.

Table 3 describes the simple average (descriptive analysis) obtained in each group (TG and CG) in the different farms. Comparing the simple means of the CG (48.58%) and the TG (58.34%), a 9.76 percentage point difference was obtained between the groups. We are aware that the pregnancy rate can be interfered with by several factors or variables, going well beyond “just the location of the farm”. So, we proposed to discuss based on a statistical model, which considers “product dose-effect” within contemporary groups, grouping all impacting variables for “pregnancy rate” within each of the YG created. There were so many variables that 107 YG were created in the established statistical model. Notably, among the calves born in this experiment, more than 900 of them conceived and gestated in the uterus that had contact with the eGAL-1 protein (dams belonging to the TG group) during the FTAI procedure. No congenital defects or stillbirths were observed in cows that received the eGAL-1 dose.

## 4. Discussion

### 4.1. Efficacy of a Single Dose of eGAL-1 on the Increase in Pregnancy Rate

The results showed that the study’s objective was achieved, demonstrating the item’s efficacy under test. An increase in pregnancy rate of 8.68 percentage points was observed (comparing treated versus control animals within the same contemporary group assessed by the statistical model) when a single dose of eGAL-1 was combined with the FTAI technique.

As is known, several factors interfere with the pregnancy rate. Therefore, the material and methods described in this article were exhaustively detailed, aiming to increase the robustness of the study and try to prove the hypothesis; hence, the use of a statistical model considers the distribution in YG. Important points inherent to this issue are that: the YG were built before the stage of exclusion of matrices due to body score, a fact that explains the difference between the number of animals in the treated and control groups; the particularities of sanitary and nutritional management of the different batches of animals worked are perfectly distributed in the statistical model based on the grouping by YG; the negative effect of nutritional deficiency on pregnancy rate (or increased probability of pregnancy loss) was controlled in the experiment, excluding mothers who did not maintain their body score, as mentioned above.

Thus, the pregnancy rate obtained on TG was different (in this case, 8.68% higher), only because of the eGAL-1 administration (*p* < 0.0001). Yet, remember that the YG effect, under binomial distribution (pregnant or not pregnant), was not statistically significant (*p* = 17.87). However, in the scenery with 3469 dams, 3 animal categories, 46 different bulls, 23 inseminators, several batches, and 2 treatment groups, it is reasonable to consider the YG effect as a biological effect. The recommendation to use a dose of eGAL-1 during an FTAI procedure was reasonable in the beef cattle routine. On average, when we administrate de eGAL-1, the whole procedure spent only 6 ± 5 s more than the conventional procedure—ten seconds as a price to obtain 8.68% more chance to impregnate a dam—is reasonable in the animal production systems.

Since 2008, the authors have worked on this development regarding establishing a manufacturing process which is consolidated, robust on homogeneity, stability, and efficiency. Since then, there have been more than 12 thousand instances of AI procedures of bovine dams, and there were no undesired biological effects—there were no reports of discomfort, pain, and irritation with the administration of the product, except for those already known in artificial insemination procedures. It is also important to note that stillbirths, malformations, and/or neonatal complications were not verified in animals that had contact with the exogenous protein. There are no reports of intoxication in humans using GAL-1 as an active drug ingredient. In the current literature, galectins (soluble in blood serum or expressed in tissues) have generally been used as biological markers of several pathological events [13,14,15], directing treatments, but still in experimental stages.

### 4.2. Why Could Galectin-1 Improve the Pregnancy Rate?

Galectins are a family of evolutionarily conserved proteins distributed from lower invertebrates to mammals [16,17]. Thus, the efficacy of recombinant human Gal-1 under assisted reproduction procedures was evaluated, besides bovine females (this work), ovine, and equine (unpublished data). All evaluations presented in species different from bovine, promising results only with dosage adjustment because of the area (cm^2^) of lumen uterus of each species, and it can include the human species here.

Galectins are multifunctional molecules that participate in several biological processes such as adhesion, proliferation, cell cycle, apoptosis, RNA processing, control of the inflammatory process, and physiological mechanisms of reproduction [9,18,19,20,21,22,23,24,25].

The galectin’s maternal–fetal tolerance role, both innate and adaptive, is associated with regulating and modulating the embryo elongation events’ immunological responses and adherence to the endometrium. Besides GAL-15 and GAL-1, other galectins can be expressed by mammals’ endometrium and the placenta. They present essential functions in differentiating the endometrium, implanting the blastocyst, and differentiating the trophoblast [26]. They contribute to placentation, regulating the development, migration, and trophoblastic invasion, all events essential to early gestational development [9,24,25,27,28]. They even act in the maternal immunological tolerance mechanism to fetal alloantigens, regulating the natural killer uterus cells and modulating T cells, mainly responsible for cellular immunity [10]. According to Blois et al. (2019) [9], the functions of Lgals1 in the context of pregnancy are the best characterized when compared to other members of the galectin family.

The endometrial expression of GAL-1 fluctuates during the estrous cycle of different phases because steroidal hormones influence it. GAL-1 has been detected in 3- to 5-day-old human embryos, acting on trophoblasts’ differentiation in the fetus’s placenta and internal cell mass [9]. The interaction between GAL-1 and integrins suggested participation at extracellular matrix and placentation events, like in the oxygen and/or nutrients exchanges or angiogenesis events (vessels formation). It can be elucidated that GAL-1 plays a vital role in maternal–fetal interface signaling since it has multiple biological functions [9,29].

Blois et al. (2007) [25] demonstrated high pregnancy loss rates in mice in which the *Lgals1* gene was deficient (knockout mice). When treating deficient mice with recombinant GAL-1, there was a decrease in fetal loss and the restoration of tolerance through several mechanisms, including the induction of tolerogenic dendritic cells, which promoted the expansion of regulatory T cells secreting interleukin-10 (IL-10) in vivo. Consequently, the protective effects of GAL-1 have been revoked in mice depleted of regulatory or IL-10 deficient T cells. Thus, Blois et al. (2007) [25] demonstrated the fundamental importance of GAL-1 in fetomaternal tolerance and the synergy between GAL-1 and progesterone in maintaining pregnancy. This article can confirm the involvement of GAL-1 in the pregnancy determination and evolution, even the massive amount of interference factors on the pregnancy that we tried to control by YG creating.

The efficacy of the technology should also be observed carefully. If it has not been performed consistently, considering the experimental and statistical model (as described in Section 2.5.5.), the effect of effectiveness can be masked. Thus, when used correctly, the results are promising. In cattle, a single eGAL-1 intrauterine dose is indicated as a health catalyst of animal fertility, it being, therefore, a tool to increase the reproductive/productive efficacy and the economic profitability using the eGAL-1 was discussed by Roncoletta et al. (2021) [29]. The approach can be different in humans and endangered mammals, not for economic appeal, but as an alternative method to aid in the exhausting process of helping some couples achieve pregnancy.

## 5. Conclusions

This study showed the single-dose eGAL-1’s effectiveness in improving the beef cattle pregnancy rate. The procedure may take 5 to 10 s longer than the conventional procedure; however, the statistics show a considerable increase in the pregnancy rate. Considering the “eGAL-1 administration effect”, it is possible to improve 8.68% of the chances of pregnancy in an inseminated cow. The exogenous protein administration was demonstrated to be safe for the female, fetus, and neonates since there were no reports of discomfort, pain, and irritation with the administration of the eGAL-1, except for those already known in artificial insemination procedures. It is also important to note that stillbirths, malformations, and/or neonatal complications were not verified.

## 6. Patent

The company, assisted by specialized lawyers, has already patented this innovation in several countries due to this highly innovative and unique technological content. This patent was co-participated with the University of São Paulo (FFRP). World patent WO/2012/083396.

## Figures and Tables

**Table 1:** The Estrus Synchronization Protocols adopted by the partner farm (in letter codes) dependently on the cows’ category (H = heifers, P = primiparous, or M = multiparous). Days of hormones administration (D0 = day zero, D7 = day seven, D8 = day eight, D9 = day nine) and TFAI day (day of the AI procedure (with or without the eGAL-1 administration)).

Farms Codes	Category	D0	D7	D8	D9	TAI Day
A	P	EB 2.0 mL	-	-	PGF_2α_ 2.0 mL ECP 1.0 mL eCG 1.5 mL	D11
K	M	EB 2.0 mL	-	-	PGF_2α_ 2.0 mL ECP 1.0 mL eCG 1.5 mL	D11
B	M	EB 2.0 mL	-	PGF_2α_ 1.5 mL ECP 0.5 mL eCG 1.5 mL	-	D10
C	M	EB 2.0 mL	PGF_2α_ 2.0 mL	-	ECP 0.5 mL eCG 1.5 mL	D11
F J L K M N O	H	EB 2.0 mL	-	PGF_2α_ 2.0 mL ECP 1.0 mL eCG 0.5 mL	-	D10
G H I J L M N O P Q R T	M	EB 2.0 mL	-	PGF_2α_ 2.0 mLECP 1.0 mLeCG 1.5 mL	-	D10
J	P	EB 2.0 mL	-	PGF_2α_ 2.0 mLECP 1.0 mLeCG 1.5 mL	-	D10

EB—Estradiol Benzoate. PGF2α—Prostaglandin F2alpha. EC—Estradiol Cypionate. eCG—Equine Chorionic Gonadotropin.

**Table 2:** Dose least squares means, using generalized linear model (GLM), with GENMOD procedure, under binomial distribution (pregnant/not pregnant) and in logarithmic function (PROBIT), by source as “dose of eGAL-1”, that means dose 0 = GC and dose 200 = TG, using SAS software, Version 6.9.

Dose	SE	Mean	SE of Mean
0	0.07005	0.494 ^a^	0.01751
200	0.06969	0.580 ^b^	0.01697

SE = Standard error; mean = probability of success of pregnancy rate; (d) SE of means = standard error of the probability of average pregnancy rate. ^a^,^b^ differ by statistics analysis method (*p* < 0.0001).

**Table 3:** Average of pregnancy rate and the number of cows on the control group (%PCG and nCG) and treatment Group (%PTG and nTG) by each farm (identified by code name) and by all farms. The font in bold format indicates a higher %P in TG.

	Groups			
	CG		TG	
Code Name of Farms	nCG	%PGC	nTG	%PTG
A	52	38.46	54	**48.15**
B	100	50.00	617	**60.62**
C	169	46.75	281	**57.65**
F	18	50.00	19	**73.68**
G	25	60.00	20	**70.00**
H	22	59.09	20	**85.00**
I	29	62.07	18	61.11
J	337	47.48	215	**53.95**
K	80	55.00	82	50.00
L	274	50.36	197	**56.35**
M	42	40.48	24	**45.83**
N	74	47.30	65	46.15
O	66	57.58	66	**65.15**
P	90	35.56	90	**53.33**
Q	45	37.78	40	**65.00**
R	92	55.43	93	**68.00**
ALL	1515	48.58	1901	58.34

## Abbreviations

AI	artificial insemination
FTAI	fixed-time artificial insemination procedure
eGAL-1	exogenous GAL-1 dose, human recombinant galectin-1
YG	contemporary groups
ET	embryo transfer
TG	treated group
CG	controlled group
BSC	body condition score
GAL-1	galectin 1
GAL-15	Galectin-15
CG	Control group
nCG	Number of cows in the control group
TG	Treated group
nTG	Number of cows in the treated group
